# Stability of milk fatty acid profile during simulated shipping for analysis by gas chromatography

**DOI:** 10.3168/jdsc.2020-0072

**Published:** 2021-06-03

**Authors:** A.J. Schmidt, R. Bomberger, K.J. Harvatine

**Affiliations:** Department of Animal Science, Penn State University, University Park 16802

## Abstract

Milk fat *trans-*10 C18:1 can be used in diagnosing low milk fat production on dairy farms because it is a specific marker of biohydrogenation-induced milk fat depression. Individual fatty acids (FA), including the *trans* C18:1 isomer, can be determined only by gas-liquid chromatography. The analysis is currently available at a limited number of laboratories and often requires long-distance shipping. Expedited shipping with dry ice or ice packs is expensive. Therefore, the objective of this study was to determine the effect of heat treatment before shipping, shipping temperature, and shipping time on milk FA profile. Samples were collected from 3 farms on 2 occasions and stored in a polystyrene foam cooler with an ice pack, at room temperature, or at 37°C for 1, 2, 3, and 7 d. Heating the sample before shipping, shipping temperature, and shipping time had very little effect on any FA analyzed. Differences observed were of small magnitude and not of practical importance, demonstrating that milk FA profile is expected to be very stable during shipping. Based on this, we propose that freezing samples and shipping in a sealed bag by second-day shipment is appropriate and demonstrated that this had little effect on FA profile of 48 milk samples. Importantly, these methods are recommended only for gas-liquid chromatography analysis of FA profile. Freezing is not appropriate for shipping for analysis by mid-infrared spectrometry-based methods or methods quantifying compounds per unit of milk because it is difficult to homogenize samples after freezing or extended shipping that results in denaturization of proteins and breaking of fat globules.

## Highlights

• Milk fatty acid profile was very stable at all storage temperatures.
• Shipping on dry ice is not required for fatty acid analysis by gas-liquid chromatography.
• Importantly, this conclusion is specific for gas-liquid chromatography analysis and not other infrared spectrometry-based methods.

## Graphical Abstract

**Figure:**
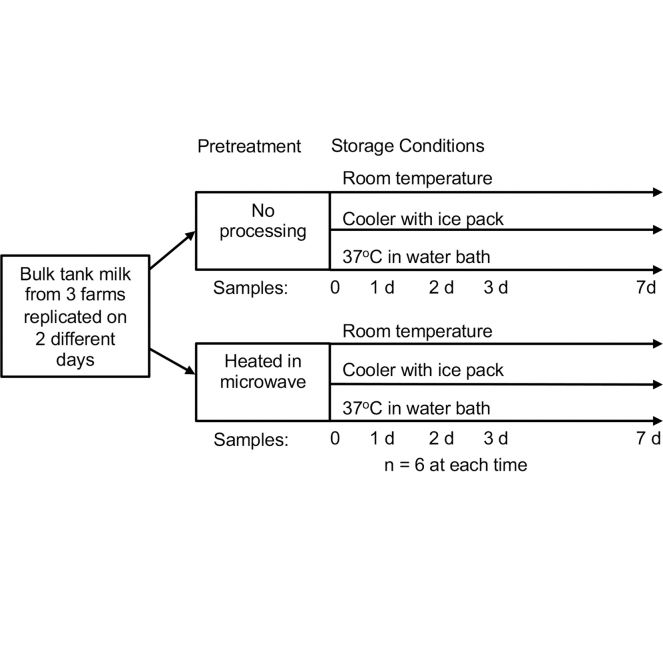
**Summary:** Milk fat *trans*-10 C18:1 can be used in diagnosing milk fat depression, but it must be determined using gasliquid chromatography that commonly requires long-distance shipping for analysis. The objective of this study was to determine the effect heat treatment before storage and different storage temperatures on milk fatty acid (FA) profile over time to recommend a suitable shipping method. Heating the sample before shipping, shipping temperature, and shipping time had very little effect on any FA analyzed. Based on this, we propose that freezing samples and shipping in a sealed bag by second-day shipment is appropriate for gas-liquid chromatography analysis of FA profile.

Optimizing nutrition and management for maximal milk fat yield is economically important on dairy farms. Milk fat yield is affected by many factors, including diet, which has a large effect (Harvatine et al., 2009). Biohydrogenation-induced milk fat depression occurs during altered rumen fermentation and results in bioactive *trans-*10 intermediates produced in the rumen that decrease milk fat synthesis. These intermediates are incorporated into milk fat and have been proposed as a tool to diagnose milk fat depression. Matamoros et al. (2019) recently reported a meta-regression of the relationship between *trans-*10 C18:1 and expected milk fat yield, providing guidelines for expected concentrations. Total de novo fatty acids (**FA**) have also been correlated with milk fat concentration (Woolpert et al., 2016; Matamoros et al., 2019) and odd- and branched-chain FA that are synthesized by rumen microbes and in the mammary gland from odd- and branched-chain VFA and have been correlated with changes in rumen microbial populations and rumen fermentation patterns (Vlaeminck et al., 2006).

Gas-liquid chromatography provides a highly accurate and precise direct quantification of milk FA profile, including *trans* isomers, and recently became commercially available at some traditional forage testing laboratories. Because GLC analysis of FA requires specific chemical preparation and expensive instrumentation, it is available at only a few centralized laboratories and commonly requires long-distance shipping. Traditionally, milk samples for FA analysis by GLC are shipped frozen and packed with dry ice by next-day delivery, with shipping sometimes costing more than the FA analysis.

Bronopol is an antimicrobial compound traditionally used to preserve milk for analysis of milk fat and protein concentration by mid-infrared spectrometry. The effect of bronopol preservative on milk FA profile after 24 h at 4°C has been investigated, but not over longer shipping periods (Butler and Stergiadis, 2011). Interestingly, bronopol had an effect on the concentration of some long-chain FA that are in low abundance. Additionally, although bronopol is routinely used in DHIA testing, it is not commonly stocked on farms or carried by nutritionists and farm consultants. It was not tested in the current study because of the previously reported effect on FA profile and the additional barrier of availability.

The objective of this study was to determine the effect of shipping temperature and shipping time on the stability of milk FA profile. We also tested the effect of a microwave heat-treatment method on FA stability during shipping. Our hypothesis was that milk FA profile would be stable for 2 to 3 d at room temperature and that heat pretreatment would extend shipping stability, allowing utilization of lower cost 2- to 3-d ground shipping.

Fresh milk samples were collected twice from bulk tanks at 3 dairy farms in central Pennsylvania. A portion of each sample was heated to approximately 77°C in a ceramic drinking mug using a conventional consumer microwave to provide the heat-treatment intervention. This temperature is very similar to the temperature to which most people would heat coffee or tea in a microwave and was selected because it is easy to describe and conduct. For comparison, the Grade “A” Pasteurized Milk Ordinance (FDA, 2017) defines batch pasteurization as 63°C held for 30 min.

The effect of shipping temperature and time was investigated in both the raw and heat-treated milks (see Graphical Abstract). Samples were stored in 50-mL screw-top polypropylene tubes (Corning Falcon 50-mL conical-bottom nonsterile centrifuge tubes, Fisher Scientific). The 2 shipping scenarios were shipping in a polystyrene foam cooler at room temperature or shipping in a polystyrene foam cooler with ice packs included at the start of the experiment. A third positive control for microbial growth was incubation in a 37°C water bath. The coolers were approximately 24 × 26 × 15 cm (inside dimension; volume: ~9,400 cm^3^) with an approximately 8-cm wall thickness and were stored at room temperature during the experiment. Ice packs were approximately 1.9 L of water frozen in self-sealing bags. The trial was replicated on 2 occasions on each of the 3 farms 8 d apart, with 1 sample per time point per farm in each run. This replication allowed parameterization of the herd effect that encompasses many things, including microbiome effects. Incubations were terminated at 1, 2, 3, and 7 d and immediately frozen at −20°C. The final data set included n = 6 samples at each time point for each treatment.

Lipids were extracted from 1 mL of stored samples at each time point using hexane:isopropanol extraction, and FA were transmethylated with sodium methoxide and analyzed using GLC with flame ionization detector (100-m SP-2560 column, Supelco Inc.) as described by Baldin et al. (2018). *Trans* isomers were identified by elution order based on previous publications. Fatty acids were reported as a percent of total FA.

Data were analyzed in JMP Pro 14 (SAS Institute Inc.). The model included the random effect of farm by sampling replicate and the fixed effects of heat treatment, shipping method, shipping time, and all 2- and 3-way interactions. The preplanned contrast tested the effect of treatment within each week. Significance was declared at *P* < 0.01.

Microwave heating had only minor effects on milk FA profile (full data available online at https://scholarsphere.psu.edu/resources/bd329dd8-350e-4a50-97a5-f5d584f7b817). The overall median coefficient of variation between methods and heating was 0.5%, and the absolute difference between control and heated milks averaged 0.015 percentage unit (median: 0.0027 percentage unit). Of specific interest, *trans*-10 C18:1 differed between control and heated milks by 0.02 percentage unit. Milk fat *trans*-10 C18:1 is expected to increase more than 0.2 percentage unit on farms experiencing mild milk fat depression; thus, the technical variation is rather small.

There was a 3-way interaction of heat pretreatment, shipping temperature, and shipping time for C14:0 (*P* < 0.001), C16:0 (*P* < 0.01), FA <16 C (*P* < 0.01), and FA >16 C (*P* < 0.01). These are the most abundant FA, and the high accuracy and precision of GLC methodology provided high power to detect these very small differences, which are not expected to be biologically important for most applications. For example, de novo synthesized FA had a range of 0.27 percentage unit between treatments (1% difference), and preformed FA had a range of 0.60 percentage unit (1.5% difference). The reported variation should be considered relative to the magnitude of differences expected.

Although some interactions of heat pretreatment and shipping time were significant (Table 1), the actual differences were very small and milk FA profiles were very stable during shipping. The average coefficient of variation was 1.3% (median: 0.7%), and the average range between treatments was 0.05 percentage unit (median: 0.01 percentage unit). *Trans-*10 C18:1 differed by 0.01 percentage unit between treatments, and de novo synthesized and preformed FA differed by 0.17 and 0.34 percentage unit between treatments, respectively, with no clear effect of heat pretreatment or time. Total odd- and branched-chain FA also were minimally affected (CV: 0.3%; range: 0.03 percentage unit). Although the heat pretreatment method is simple and has little to no effect on FA profile, it is not needed because FA profile is stable during shipping.

**Table 1:** Effect of preheating in a microwave on the profile of selected milk fatty acids (FA) during simulated shipping over 7 d^1^

FA,^2^ % of FA	Day 1		Day 2		Day 3		Day 7		SEM	*P*-value^3^		
	Con	Preheat	Con	Preheat	Con	Preheat	Con	Preheat		Day	Preheat	Int
C8:0	1.31	1.30	1.31	1.31	1.31	1.32	1.29	1.31	0.023	<0.001	0.13	<0.001
C10:0	2.90	2.89	2.90	2.90	2.90	2.91	2.86	2.90	0.086	<0.01	0.25	<0.01
*cis*-9 C10:1	0.25	0.25	0.25	0.25	0.25	0.25	0.25	0.25	0.013	<0.001	0.62	<0.01
C14:0	10.5	10.5	10.5	10.5	10.5	10.5	10.6	10.5	0.236	0.25	0.57	<0.001*
C15:0	1.03	1.03	1.03	1.03	1.03	1.03	1.04	1.03	0.040	0.12	0.69	<0.01
C16:0	28.0	28.0	27.9	27.9	28.0	27.9	28.1	27.8	0.907	0.09	0.33	<0.001*
C17:0	0.58	0.58	0.58	0.58	0.58	0.58	0.59	0.58	0.027	<0.001	0.56	<0.001
*trans*-10 C18:1	0.49	0.49	0.49	0.49	0.49	0.49	0.49	0.50	0.102	0.04	0.86	0.99
*trans*-11 C18:1	1.53	1.53	1.54	1.53	1.54	1.54	1.54	1.53	0.300	0.04	0.96	0.74
*cis*-9 C18:1	18.9	18.8	18.8	18.9	18.7	18.9	18.6	18.9	0.485	<0.01	0.47	<0.001
*cis*-9,*cis-*12 C18:2	2.44	2.44	2.43	2.45	2.41	2.45	2.38	2.45	0.245	<0.01	0.95	<0.001
*cis-*9*,cis-*12,*cis-*15 C18:3	0.68	0.68	0.63	0.69	0.67	0.69	0.67	0.68	0.109	0.58	0.94	0.53
*cis-*9,*trans-*11 CLA	0.66	0.66	0.68	0.68	0.65	0.66	0.65	0.66	0.119	<0.001	0.19	0.03
By source^4^												
<16	26.1	26.0	26.1	26.1	26.0	26.1	26.0	26.0	0.52	<0.01	0.02	0.09
16C	29.2	29.3	29.2	29.2	29.2	29.1	29.3	29.1	1.00	0.09	0.32	<0.001*
>16C	39.3	39.3	39.2	39.4	39.2	39.4	39.1	39.4	1.60	0.74	0.43	<0.001*
OBCFA	3.65	3.64	3.65	3.64	3.66	3.65	3.68	3.66	0.207	<0.001	0.63	0.49

1 Milk was stored raw (Con) or after being heated to approximately 77°C in a microwave (Preheat). Samples were collected from 3 farms on 2 occasions (n = 6/treatment). Samples were stored at room temperature, in a cooler with an ice pack, or in a 37°C water bath; however, shipping method interactions were of a very small magnitude and not relevant to this field application.

2 Full profile available online at https://scholarsphere.psu.edu/resources/bd329dd8-350e-4a50-97a5-f5d584f7b817.

3 Interaction (Int) of preheating (Preheat) and shipping time (Day; significance declared at *P* < 0.01).

4 <16C = sum of even-carbon straight-chain FA that originate from de novo synthesis in the mammary gland; 16C = sum of 16-carbon FA that originate from both de novo synthesis and uptake of preformed FA from plasma; >16 C = sum of FA >16 carbons that originate from plasma; OBCFA = sum of identified odd- and branched-chain FA.

* Indicates 3-way interaction of heat pretreatment, shipping temperature, and shipping time (*P* < 0.01; all very small and not relevant to field application).

There was also little effect of shipping method on FA profile because it was stable even when shipping at 37°C (Table 2). Overall, the coefficient of variation between treatment means across days averaged 1.5% (median: 0.8%), and the average range between treatment means was 0.05 percentage unit (median: 0.01 unit). Of specific interest, *trans-*10 C18:1 was not affected by shipping method or shipping time and differed by 0.02 percentage unit across the experiment. An interaction of shipping method and time was observed for some specific FA, but the magnitude of the change was very small and not expected to be relevant to field application (Table 2).

**Table 2:** Effect of storage temperature and time on profile of selected milk fatty acids (FA) during simulated shipping over 7 d^1^

FA,^2^ % of FA	Day 1			Day 2			Day 3			Day 7			SEM	*P*-value^3^		
	Ice pack	RMT	37°C	Ice pack	RMT	37°C	Ice pack	RMT	37°C	Ice pack	RMT	37°C		Day	Temp	Int
C14:0	10.5	10.5	10.5	10.5	10.5	10.5	10.5	10.5	10.5	10.6	10.5	10.5	0.24	0.25	0.51	<0.001
*anteiso* C15:0	0.41	0.41	0.41	0.41	0.42	0.41	0.41	0.42	0.41	0.42	0.42	0.41	0.039	<0.001	0.51	<0.001
*cis*-9 C14:1	0.78	0.78	0.78	0.77	0.79	0.77	0.78	0.78	0.78	0.77	0.77	0.77	0.044	0.01	0.65	<0.001
C15:0	1.03	1.03	1.03	1.03	1.03	1.03	1.03	1.03	1.03	1.04	1.03	1.03	0.040	0.12	0.05	<0.001
*iso* C16:0	0.23	0.23	0.23	0.23	0.23	0.23	0.23	0.23	0.23	0.23	0.24	0.23	0.028	<0.001	0.99	<0.001
*cis-*9 C16:1	1.24	1.24	1.25	1.24	1.25	1.24	1.25	1.24	1.24	1.23	1.24	1.25	0.097	0.63	0.01	<0.01
C18:0	11.7	11.7	11.6	11.6	11.5	11.7	11.6	11.7	11.6	11.7	11.7	11.7	0.66	<0.01	0.07	<0.001
*trans*-10 C18:1	0.49	0.49	0.49	0.49	0.49	0.48	0.48	0.49	0.49	0.49	0.50	0.49	0.10	0.04	0.86	0.49
*trans*-11 C18:1	1.53	1.53	1.53	1.53	1.53	1.55	1.54	1.54	1.54	1.53	1.54	1.53	0.30	0.04	0.76	0.42
*trans*-15 C18:1	0.33	0.34	0.34	0.34	0.34	0.35	0.35	0.36	0.35	0.34	0.32	0.31	0.032	<0.001	0.11	<0.001
*cis*-11 C18:1	0.62	0.62	0.62	0.62	0.62	0.62	0.62	0.62	0.63	0.62	0.61	0.61	0.066	<0.001	0.88	<0.01
*cis-*12 C18:1	0.36	0.36	0.36	0.36	0.36	0.35	0.35	0.36	0.36	0.36	0.36	0.36	0.073	0.58	0.85	<0.05
*cis-*9,*cis-*12 C18:2	2.43	2.43	2.45	2.43	2.46	2.43	2.43	2.43	2.43	2.40	2.43	2.43	0.25	<0.01	0.04	0.02
*cis-*9,*cis-*12,*cis-*15 C18:3	0.68	0.68	0.69	0.61	0.69	0.68	0.68	0.68	0.68	0.67	0.68	0.68	0.11	0.58	0.97	0.48
*cis-*9,*trans-*11 CLA	0.66	0.66	0.67	0.68	0.68	0.67	0.67	0.65	0.66	0.65	0.65	0.65	0.12	<0.001	0.06	0.02
C20:5 n3	0.054	0.055	0.055	0.055	0.054	0.056	0.056	0.055	0.054	0.052	0.054	0.055	0.011	0.07	0.80	<0.01
Sum by source^4^																
<16 C	26.0	26.0	26.0	26.0	26.1	26.1	26.1	26.1	26.1	26.0	26.0	26.0	0.52	<0.01	0.57	0.37
16 C	29.3	29.3	29.2	29.2	29.2	29.1	29.2	29.2	29.1	29.4	29.1	29.1	1.00	0.09	0.02	0.01
>16 C	39.3	39.3	39.4	39.2	39.3	39.4	39.3	39.3	39.3	39.1	39.3	39.4	1.61	0.74	0.23	0.15
OBCFA	3.65	3.64	3.65	3.64	3.66	3.65	3.65	3.66	3.66	3.67	3.67	3.67	0.21	<0.001	0.89	0.44

1 Samples were collected from 3 farms on 2 occasions (n = 6/treatment). Samples were stored in a cooler with an ice pack, at room temperature (RMT), or in a 37°C water bath. Samples were also stored either raw or after being heated to approximately 77°C in a microwave; however, heat pretreatment interactions were of a very small magnitude and not relevant to this field application.

2 Full profile available online at https://scholarsphere.psu.edu/resources/bd329dd8-350e-4a50-97a5-f5d584f7b817.

3 *P*-values for the main effect of time (Day) and storage temperature (Temp) and their interaction (Int; significance declared at *P* < 0.01).

4 <16C = sum of even-carbon straight-chain FA that originate from de novo synthesis in the mammary gland; 16C = sum of 16-carbon FA that originate from both de novo synthesis and uptake of preformed FA from plasma; >16 C = sum of FA >16 carbons that originate from plasma; OBCFA = sum of identified odd- and branched-chain FA.

Based on the small difference in FA profile, a conservative recommendation is to freeze samples and ship for second-day delivery. To validate this recommendation, a daily composite of milk was collected from 48 cows, and milk fat was extracted from the milk raw and after freezing and storing in a polystyrene foam cooler for 2 d to simulate shipping. There was no effect on *trans-*10 C18:1 or *trans-*11 C18:1, although very small differences were observed in *cis-*9,*cis-*12 C18:2 (0.05-unit decrease), *cis-*9,*cis-*12,*cis-*15 C18:3 (0.09-unit decrease), de novo synthesized FA (0.33-unit decrease), and total 16C FA (0.63-unit increase; Table 3).

**Table 3:** Effect of freezing and storage in a foam cooler simulating shipping over 2 d on milk fatty acid (FA) profile

FA,^1^ % of FA	Treatment^2^		SEM	*P-*value^3^
	Raw	Frozen		
*trans-*10 C18:1	0.56	0.55	0.04	0.60
*trans*-11 C18:1	1.32	1.31	0.05	0.36
*cis-9* C18:1	20.1	19.7	0.29	<0.01
*cis-*9,*cis-*12 C18:2	2.81	2.76	0.04	0.01
*cis-*9,*cis-*12,*cis-*15 C18:3	0.66	0.57	0.01	<0.01
*cis-*9,*trans-*11 CLA				
Sum by source^4^				
<16 C	26.2	25.9	0.30	0.02
16 C	28.7	29.3	0.40	<0.01
>16 C	40.3	40.2	0.48	0.54
OBCFA	2.95	2.94	0.04	0.72

1 Full profile available online at https://scholarsphere.psu.edu/resources/bd329dd8-350e-4a50-97a5-f5d584f7b817.

2 Samples were collected from 48 cows. Milk fat was either extracted immediately from raw milk (Raw) or extracted from milk that was frozen and then stored for 2 d in a polystyrene foam cooler to simulate shipping (Frozen).

3 Difference between Raw and Frozen treatments.

4 <16C = sum of even-carbon straight-chain FA that originate from de novo synthesis in the mammary gland; 16C = sum of 16-carbon FA that originate from both de novo synthesis and uptake of preformed FA from plasma; >16 C = sum of FA >16 carbons that originate from plasma; OBCFA = sum of identified odd- and branched-chain FA.

Overall, the current study demonstrated that FA profile is very resistant to modification and degradation during shipping and provided the data for individuals to select the best shipping method for their situation. In most situations, the cost of expedited shipment with dry ice or ice packs is likely not justified. It is important to note that incubation at 37°C resulted in popping of the snap caps on the vials. Based on previous experience, snap-cap and screw-top vials will open during shipment if not packed well due to shaking during transport. Taping vial tops and placing vials in sealed bags (individually if possible) are good practices. If shipping is delayed for extended periods of time, milk FA profile is not expected to be affected, although sample vials may explode and be lost if not in a sealed bag. Importantly, freezing and extended storage time will cause clumping and difficulty in homogenizing samples and are not suitable for analysis by mid-infrared spectrum analysis or assays determining absolute concentration in milk.

In conclusion, milk FA profile is highly stable during shipping even at high temperature before GLC analysis. When shipping a limited number of samples, we recommend freezing milk, placing each vial in a sealed bag, and shipping by second-day delivery as an economical approach that is more than adequately cautious in terms of sample preservation. Importantly, this method should be used only for GLC analysis of milk FA profile and not for other methods, including fast-Fourier infrared spectrometry.
